# Three-dimensional electrochemical-magnetic-thermal coupling model for lithium-ion batteries and its application in battery health monitoring and fault diagnosis

**DOI:** 10.1038/s41598-024-61526-0

**Published:** 2024-05-11

**Authors:** Xuanyao Bai, Donghong Peng, Yanxia Chen, Chaoqun Ma, Wenwen Qu, Shuangqiang Liu, Le Luo

**Affiliations:** 1 School of Physics and Astronomy, Sun Yat-Sen University, Zhuhai, China; 2 Shenzhen Research Institute of Sun Yat-Sen University, Shenzhen, China; 3 State Key Laboratory of Optoelectronic Materials and Technologies, Sun Yat-Sen University (Guangzhou Campus), Guangzhou, China; 4 International Quantum Academy, Shenzhen, China

**Keywords:** Lithium-ion battery, Magnetic field detection, Coupling model, Internal short circuit, Electrochemistry, Energy, Applied physics, Techniques and instrumentation

## Abstract

Storage batteries with elevated energy density, superior safety and economic costs continues to escalate. Batteries can pose safety hazards due to internal short circuits, open circuits and other malfunctions during usage, hence real-time surveillance and error diagnosis of the battery’s operational state is imperative. In this paper, a three-dimensional model of electrochemical-magnetic field-thermal coupling is formulated with lithium-ion pouch cells as the research focus, and the spatial distribution pattern of the physical field such as magnetic field and temperature when the battery is operational is acquired. Furthermore, this manuscript also investigates the diagnostic methodology for defective batteries with internal short circuits and fissures, that is, the operational state of the battery is evaluated and diagnosed by the distribution of the magnetic field surrounding the battery. To substantiate the method’s practical viability, the present study extends its examination to the 18650-battery pack. We obtained the magnetic field images of the normal operation of the battery pack and the failure state of some batteries and analyzed the relationship between the magnetic field distribution characteristics and the performance of the battery pack, providing a new method for the health monitoring and fault diagnosis of the battery pack. This non-contact method incurs no damage to the battery, concurrently exhibiting elevated sensitivity and extremely rapid response time. Meanwhile, it provides an effective means for non-destructive research on the batteries and can be applied to areas such as battery safety screening and non-destructive testing. This research not only helps to facilitate our understanding of the battery’s operating mechanism, but also provides robust support for safe operation and optimal battery design.

## Introduction

Lithium-ion batteries, characterized by high energy density, large power output, and rapid charge–discharge rates, have become one of the most widely used rechargeable electrochemical energy storage devices^1^. They find extensive applications in various domains such as electronic products, electric vehicles, and grid energy storage systems^2–4^. However, the lithium-ion batteries primarily consist of flammable electrolytes and active electrode materials, under high temperature abuse or accidental conditions, the batteries may undergo thermal runaway due to exothermic reactions, potentially leading to fire incidents^5,6^. To prevent accidents, it is crucial to conduct safety testing and screening on the commercial lithium-ion batteries. Currently, common detection methods for lithium-ion batteries include disassembly characterization methods and in-situ characterization methods. Disassembly methods, such as scanning electron microscopy (SEM)^7,8^, provide rich information about battery material properties. However, they may alter the internal structure during disassembly, leading to less accurate information. Moreover, invasive detection methods like disassembly can disrupt the battery structure, making it difficult to obtain accurate fault information. In-situ characterization methods, such as X-ray CT scanning^9,10^, offer non-destructive measurements, but their relatively slow scan speed and inability to reflect the chemical and physical changes within the battery limit their effectiveness^11^. Therefore, an efficient and non-destructive detection method is required for monitoring and assessing the batteries.

In recent years, a non-destructive fault detection method based on weak magnetic field measurements of lithium-ion batteries has emerged. This method was first proposed by Ilott et al. in 2018, focusing on a non-destructive approach to study the magnetic susceptibility of batteries. The method can detect battery defects, establish a relationship between the magnetic susceptibility and charge state, and respond to differences as low as 0.1 ppm (1 μT) in susceptibilities^11^. In 2020, scientists from Johannes Gutenberg University (JGU) and the Helmholtz Institute Mainz (HIM) proposed a non-contact method to detect the charging state and defects of lithium-ion batteries^12^. They used an atomic magnetometer to measure the weak induced magnetic field around lithium-ion batteries in a magnetic shielding environment, establishing a relationship between the magnetic susceptibility and the internal defects. The magnetometer in their experiment can achieve a sensitivity of 20 fT/Hz^1/2^. Observations from the measurements showed a decrease in the total magnetic field as the battery discharged. Additionally, utilizing regularized magnetic field inversion, magnetic susceptibility maps corresponding to the measured field could be generated. In 2021, researchers led by Brauchle F directly measured the current distribution in lithium-ion batteries through magnetic field imaging^13^. Using an unshielded measurement setup with anisotropic magnetoresistive (AMR) sensors, they reconstructed the battery’s current distribution based on the measured magnetic field, achieving an accuracy of 227 mA/cm^2^ at a local resolution of 4 mm^2^. In 2022, researchers led by Bason M G from the University of Sussex utilized a magnetic flux gate array to measure the external magnetic field of batteries. Utilizing electromagnetic relationships, they extrapolated the internal current distribution within the battery, facilitating the measurement of current density on the 1 nA/cm^2^ scale^14^. To assess the accuracy of magnetic field measurements, the researchers compared the magnetic field images with those predicted by a finite element model (FEM) and they found good agreement between measured and modelled fields. Their experimental results indicated that magnetic field variations could be attributed to strain and local heating. The literature mentioned inversion methods for deriving physical quantities such as current density and magnetic susceptibility for healthy batteries. However, research on the magnetic field distribution of the batteries with defects such as internal short circuits and cracks is scarce. Consequently, further research is necessary to enhance understanding of the magnetic field distribution in faulty battery scenarios.

Building upon previous research, this paper proposes a new solution for lithium-ion battery detection based on magnetic field detection. By coupling the battery’s P2D model with a magnetic field model, a lithium battery-magnetic field coupling model is introduced. This model can calculate the magnetic field distribution around the battery during charge and discharge processes. By analyzing the magnetic field distribution, the health status of the battery can be inferred, enabling the detection and localization of battery faults. The study focuses on the magnetic field distribution around batteries in the presence of internal short circuits and cracks, providing valuable insights for practical measurement and detection. In addition, the magnetic field around a commercial battery pack is measured in this work, which shows the practicability of the model to a certain extent.

## Model development

Researchers often build electrochemical models to study electrochemical problems^15^. In this section, a simplified multi-physics coupling model for batteries is constructed through the application of P2D electrochemical model theory and the Biot-Savart law.

### The P2D model

Generally speaking, models for lithium-ion batteries are primarily categorized into three major classes: electrochemical behavior models^16–18^, thermal behavior models^19–21^, and aging behavior models^22–24^. The electrochemical model is a mechanistic framework that employs reaction kinetics equations to describe the operational processes of the battery from an electrochemical perspective. One of the most representative models in this category is the pseudo two-dimensional (P2D) model^25,26^. The model, initially proposed by Doyle and Newman, is an electrochemical model for lithium-ion batteries based on the theory of porous electrodes^25^. This model employs a set of partial differential equations and algebraic equation systems to describe the diffusion and migration processes of lithium ions in the solid–liquid phases within the battery, electrochemical reactions at the solid–liquid phase interface, Ohm's law, charge conservation law, and other phenomena. It not only accurately simulates the terminal voltage characteristics of the battery under various current excitations but also enables the simulation of the distribution of lithium-ion concentrations in the solid–liquid phases, solid–liquid phase potential distribution, and various overpotentials within the battery. The P2D model for batteries considers electrochemical reactions and the transport of lithium ions within the battery, incorporating various chemical and physical processes. Due to its higher precision, it is widely applied in research on battery capacity degradation scenarios.

**Figure 1:**
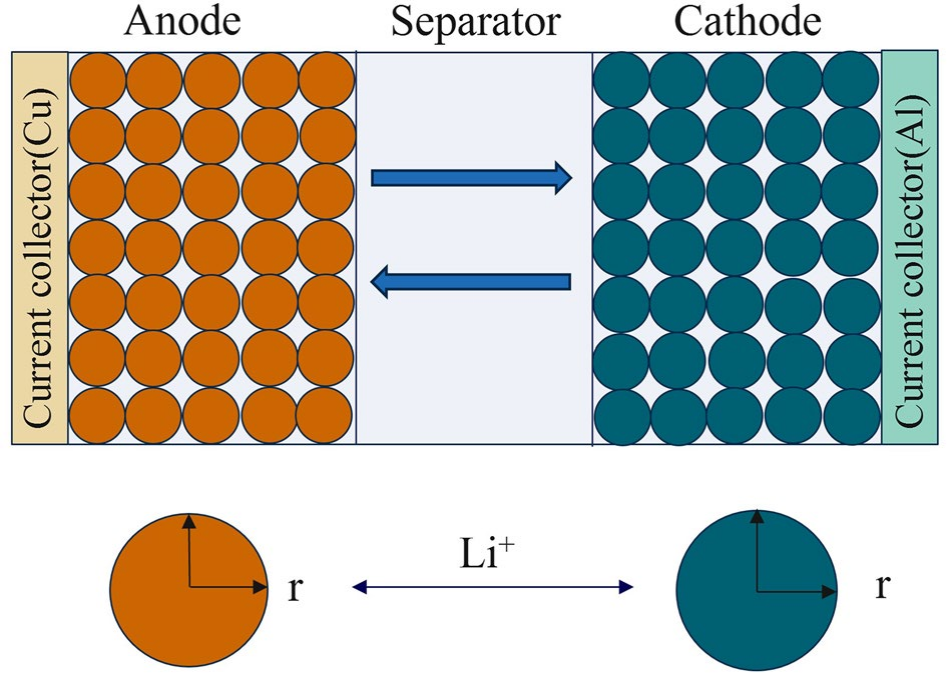
The Li-ion battery P2D model.

As shown in Fig. 1, the model posits that the battery cell comprises a positive electrode-separator-electrolyte-negative electrode assembly, in which the electrodes are porous materials and the electrolyte is in solution. The solid component consists of electroactive material particles embedded in a conductive binder matrix. The model incorporates two dimensions: the radial direction within the positive and negative electrode particles and the thickness direction of the battery electrode plates. L_n_, L_sp_, and L_p_ respectively denote the thickness of the negative electrode active material layer, separator thickness, and positive electrode active material layer thickness. The model posits that the positive and negative electrode active materials are regarded as uniformly distributed small spherical particles, and their motion is described using Fick's second law. The electrolyte is filled in both the positive and negative electrode material layers (porous electrodes) and the separator region^26,27^.

The premise of the model are two conservation principles: current conservation and mass conservation. Current conservation dictates that the total current is always equal to the sum of solid-phase current and liquid-phase current. Mass conservation ensures that the total amount of substance remains constant before and after any changes occur.

The P2D model can be described by the following equation^25,28–30^:

(1) Mass conservation for solid phase:

1 $$\frac{\partial {c}_{1}}{\partial t}=\frac{{D}_{1}}{{r}^{2}}[\frac{\partial }{r}({r}^{2}\frac{\partial {c}_{1}}{\partial r})]$$

2 $$-\nabla \cdot {c}_{1}{|}_{r=0}=0$$

3 $$-{D}_{1}\nabla {c}_{1}{|}_{r={R}_{s}}=\frac{{j}_{loc,i}}{{S}_{a,i}F}$$

where ${c}_{1}$ is the concentration of lithium in the active material, $t$ is time, ${D}_{1}$ is the diffusion coefficient of lithium in solid phase, $r$ is radius distance variable of particle, ${R}_{s}$ is the radius of electrode particles, ${j}_{loc,i}$ is local current density, ${S}_{a,i}$ is specific surface area, $F$ is Faraday’s Constant.

(2) Mass conservation for liquid phase:

4 $${\varepsilon }_{2}\frac{\partial {c}_{2}}{\partial t}=\nabla \cdot \left({D}_{2}^{eff}\nabla {c}_{2}\right)+\frac{{S}_{a,i}{j}_{loc,i}}{F}\left(1-{t}_{+}\right)$$

5 $${D}_{2}^{eff}={D}_{2}{\varepsilon }_{2}^{{\upgamma }_{2}}$$

6 $$-\nabla \cdot {c}_{2}{|}_{x=0,x=L}=0$$

7 $$-\nabla \cdot {c}_{2}{|}_{x={L}_{n}}=-\nabla \cdot {c}_{2}{|}_{x={L}_{n}+{L}_{s}}$$

where ${\varepsilon }_{2}$ is electrolyte volume fraction, ${c}_{2}$ is concentration of lithium in the electrolyte, ${D}_{2}$ is diffusion coefficient of lithium in the electrolyte, the superscript $eff$ represents valid values, ${t}_{+}$ is the transference number of Li^+^, ${\upgamma }_{2}$ is Bruggeman tortuosity factor of electrolyte, ${L}_{n}$ is the thickness of negative electrode, ${L}_{s}$ is the thickness of separator, ${L}_{p}$ is the thickness of positive electrode, $L={{L}_{n}+L}_{s}+{L}_{p}$.

(3) Charge conservation for solid phase:

8 $${i}_{1}=-{\sigma }_{1}^{eff}\nabla {\varphi }_{1}$$

9 $$\nabla \cdot {i}_{1}=-{S}_{a,i}\left({j}_{loc,i}+{C}_{dl}\left(\frac{\partial {\varphi }_{1}}{\partial t}-\frac{\partial {\varphi }_{2}}{\partial t}\right)\right)$$

10 $${S}_{a,i}=\frac{3{\varepsilon }_{1}}{{R}_{s}}$$

11 $${D}_{1}^{eff}={D}_{1}{\varepsilon }_{1}^{{\gamma }_{1}}$$

12 $$-{\sigma }_{1}^{eff}\nabla \cdot {\varphi }_{1}{|}_{x={L}_{n},x={L}_{n}+{L}_{s}}=0$$

13 $$-{\sigma }_{1}^{eff}\nabla \cdot {\varphi }_{1}{|}_{x=0}=-{\sigma }_{1}^{eff}\nabla \cdot {\varphi }_{1}{|}_{x=L}={i}_{app}$$

14 $${\varphi }_{1}{|}_{x=0}=0$$

15 $${\sigma }_{1}^{eff}={\sigma }_{1}{\varepsilon }_{1}^{{\gamma }_{1}}$$

where ${i}_{1}$ is the electronic current density in the solid phase, ${\sigma }_{1}$ is electronic conductivity of solid phase, ${\varphi }_{1}$ is solid phase potential, ${C}_{dl}$ is electrical double layer capacitance, ${\varphi }_{2}$ is solution phase potential, ${\varepsilon }_{1}$ is active material volume fraction, ${D}_{1}$ is diffusion coefficient of lithium in solid phase, ${i}_{app}$ is total applied current density.

(4) Charge conservation for liquid phase:

16 $${i}_{2}=-{\sigma }_{2}^{eff}\nabla {\varphi }_{2}+\frac{2RT{\sigma }_{2}^{eff}}{F}\left(1-{t}_{+}\right)\left(1+\frac{dlnf}{dln{c}_{2}}\right)\nabla ln{c}_{2}$$

17 $$\nabla \cdot {i}_{2}={S}_{a,i}{j}_{loc,i}$$

18 $${\sigma }_{2}^{eff}={\sigma }_{2}{\varepsilon }_{2}^{{\gamma }_{2}}$$

19 $${\nabla \varphi }_{2}{|}_{x=0,x=L}=0$$

where ${i}_{2}$ is current density in the solution phase, ${\sigma }_{2}$ is ionic conductivity of electrolyte, $R$ is gas constant (8.314 J/mol/K), $T$ is temperature of the battery.

(5) Electrochemical kinetics:

20 $${j}_{loc,i}=\frac{{i}_{0}}{F}[{\text{exp}}\left(\frac{{\alpha }_{a,i}{\eta }_{i}F}{RT}\right)-{\text{exp}}\left(\frac{{\alpha }_{c,i}{\eta }_{i}F}{RT}\right)]$$

21 $${\eta }_{i}={\varphi }_{1,i}-{\varphi }_{2},i-{U}_{oc}$$

22 $${j}_{0,i}=F{k}_{i}{c}_{2}^{{\alpha }_{an}}{\left({c}_{1,max,i}-{c}_{1,surf,i}\right)}^{{\alpha }_{an}}{c}_{1,surf,i}^{{\alpha }_{ca}}$$

where ${j}_{0,i}$ is exchanged current density, ${\alpha }_{a,i}$ is anodic transfer coefficient, ${\eta }_{i}$ is overpotential, ${\alpha }_{c,i}$ is cathodic transfer coefficient, ${U}_{oc}$ is open circuit potential, ${k}_{i}$ is reaction rate.

### Biot–Savart law

According to Maxwell’s equations and the Biot-Savart law, the magnetic field distribution can be derived from the current density distribution^13^. The current continuity equation is known as:

23 $$\nabla \overrightarrow{J}=-\frac{\partial \rho }{\partial t}$$

where $\rho$ is the charge density in this area, $t$ is the time, J is the current density. Assuming that the charge density, conductivity, and current distribution are static, Eq. (23) can be simplified as:

24 $$\nabla \overrightarrow{J}=0$$

We know:

25 $$\overrightarrow{J}=\sigma \overrightarrow{E}$$

26 $$\overrightarrow{E}=-\nabla \overrightarrow{V}$$

where $\overrightarrow{E}$ is the electric field, $\sigma$ is the conductivity, $\overrightarrow{V}$ is the electric potential. Then:

27 $$-\nabla \left(\sigma \nabla \overrightarrow{V}\right)=0$$

Then the current density can be expressed as:

28 $$\overrightarrow{J}=-\sigma \nabla \overrightarrow{V}$$

Using the resulting current distribution, the magnetic field at several measuring points around the geometry can be calculated. The magnetic field generated by an elementary current can be described by the Biot-Savart law:

29 $$\overrightarrow{H}\left(\overrightarrow{r}\right)=\frac{1}{4\pi }{\int }_{C}\frac{\overrightarrow{I}d\overrightarrow{{\ell}}\times \overrightarrow{{r}^{\prime}}}{{\left|\overrightarrow{{r}^{\prime}}\right|}^{3}}$$

where $\overrightarrow{H}\left(\overrightarrow{r}\right)$ is the magnetic field at position $\overrightarrow{r}$, $d\overrightarrow{{\ell}}$ is a vector describing a wire element along path $C$ in the direction of the current. When the ratio $|d\overrightarrow{{\ell}}|$ to $|\overrightarrow{{r}^{\prime}}|$ is small enough, the Biot-Savart law can be expressed as:

30 $$\overrightarrow{H}\left(\overrightarrow{r}\right)=\frac{1}{4\pi }\sum_{k=1}^{N}\frac{\overrightarrow{{I}_{k}}d\overrightarrow{{{\ell}}_{k}}\times \overrightarrow{{r}_{k}^{\prime}}}{{\left|\overrightarrow{{r}_{k}^{\prime}}\right|}^{3}}$$

where $N$ is the number of discretized source elements. Considering discrete measuring point $\overrightarrow{{{\varvec{H}}}_{{\varvec{i}}}}={({H}_{i,x},{H}_{i,y},{H}_{i,z})}^{T}$, the magnetic field at the $k$ th point can be expressed as:

31 $$\overrightarrow{{H}_{i}}=\frac{1}{4\pi }\sum_{k=1}^{N}\frac{\overrightarrow{{I}_{k}}d\overrightarrow{{{\ell}}_{k}}\times \left(\overrightarrow{{r}_{i}}-\overrightarrow{{{\ell}}_{k}}\right)}{{\left|{r}_{i}-\overrightarrow{{{\ell}}_{k}}\right|}^{3}}$$

The cross-product part can be simplified to:

32 $$\overrightarrow{I}\times \overrightarrow{{r}^{\prime}}=\left(\begin{array}{c}{I}_{x}\\ {I}_{y}\\ {I}_{z}\end{array}\right)\times \left(\begin{array}{c}{r}_{x}^{\prime}\\ {r}_{y}^{\prime}\\ {r}_{z}^{\prime}\end{array}\right)=\left(\begin{array}{c}{{I}_{y}r}_{z}^{\prime}\\ {{-I}_{x}r}_{z}^{\prime}\\ {{I}_{x}r}_{y}^{\prime}-{I}_{y}{r}_{x}^{\prime}\end{array}\right)$$

Then:

33 $$\overrightarrow{{H}_{i}}=\frac{1}{4\pi }\sum_{k=1}^{N}{{\varvec{g}}}_{{\varvec{i}},{\varvec{k}}}\left(\begin{array}{c}{I}_{y,k}\\ {I}_{x,k}\end{array}\right)=\frac{1}{4\pi }{{\varvec{g}}}_{{\varvec{i}}}\overrightarrow{I}$$

where

34 $${{\varvec{g}}}_{{\varvec{i}}}=\left({g}_{i,1},{g}_{i,2}\dots {g}_{i,N}\right)$$

35 $${{\varvec{g}}}_{{\varvec{i}},{\varvec{k}}}=\frac{1}{{\left|\overrightarrow{{r}_{i}}-\overrightarrow{{{\ell}}_{k}}\right|}^{3}}\left(\begin{array}{c}{r}_{z}^{\prime}\\ {-r}_{z}^{\prime}\end{array}\right)$$

36 $$\overrightarrow{I}={\left({I}_{1},\dots {I}_{N}\right)}^{T}={\left(\left({I}_{y,1},{I}_{x,1}\right)\dots \left({I}_{y,N},{I}_{x,N}\right)\right)}^{T}$$

Represent all measurements and source points in a linear system:

37 $$\left[\begin{array}{c}\begin{array}{c}{H}_{1}\\ {H}_{2}\\ \dots \end{array}\\ {H}_{M}\end{array}\right]=\frac{1}{4\pi }\left[\begin{array}{c}\begin{array}{c}{g}_{\mathrm{1,1}} \dots {g}_{1,N}\\ {g}_{\mathrm{2,1}} \dots {g}_{2,N}\\ \dots \end{array}\\ {g}_{M,1} \dots {g}_{M,N}\end{array}\right]\left[\begin{array}{c}\begin{array}{c}{I}_{1}\\ {I}_{2}\\ \dots \end{array}\\ {I}_{M}\end{array}\right]$$

Then:

38 $$\overrightarrow{H}=\frac{1}{4\pi }{\varvec{G}}\overrightarrow{I}$$

In Eq. (38), the vector $\overrightarrow{H}$ represents the magnetic field at the measurement point, while the matrix ${\varvec{G}}$ reflects the position relationship between the measurement point and the current sources, enabling the connection between current and magnetic field. This equation is derived through Biot–Savart law, which is applicable not only to calculate the magnetic field generated at any point in space but also to calculate the magnetic field distribution in positive and negative electrodes, current collectors and other structures of a battery.

### The cell model

**Figure 2:**
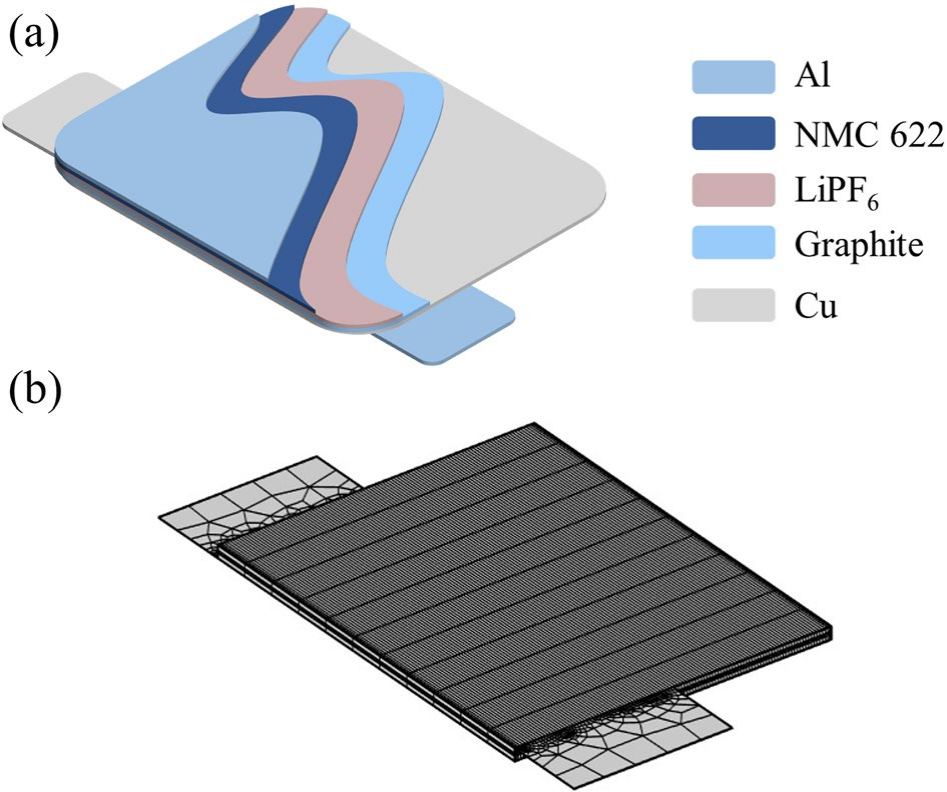
(**a**) Internal structure diagram of a lithium-ion pouch cell. (**b**) Mesh generation of the cell model.

This paper establishes a multi-physics coupling model for the commercial nickel–cobalt-manganese graphite/lithium-ion battery (NMC 622). As in Fig. 2(a), this schematic illustrates the geometric structure of a three-dimensional battery model. The battery model represents a pouch cell unit with dimensions of 5 cm in length, 4 cm in width, and 160 μm in height. The cell unit consists of a positive current collector, a positive electrode, a separator, negative electrode, and a negative current collector from top to bottom, separated by a 30 μm-thick separator. The materials and thicknesses of these components are specified as shown in Table 1. For visualization purposes, all plots are scald 10 times in the z direction due to the high aspect ratio of the geometric features. In the computation of the magnetic field, the external boundary of the cell is a rectangular air domain measuring 0.06 m × 0.09 m × 480 μm, employed to calculate the magnetic field distribution in three-dimensional space. The mesh division of the cell model is illustrated in Fig. 2b. This work is a simplified model focusing solely on the distribution of physical fields within the geometric components of the battery; therefore, the actual wiring of the battery during usage is not considered. The internal parameters used in this model are sourced from literature^31–33^ or the COMSOL built-in material library.

**Table 1:** Basic parameters of the cell.

Name	Materials	Thickness(μm)
Positive current collector	Al	5
Positive electrode	NMC 622	60
Separator	LiPF_6_	30
Negative electrode	Graphite	60
Negative current collector	Cu	5

## Results and discussion

This work establishes a lithium-ion cell model by coupling a P2D battery model with a magnetic field model. The model characterizes electrochemical quantities such as voltage distribution within the lithium-ion battery and the corresponding magnetic field distribution of the cell. In this section, we examine the magnetic field distribution associated with internal short circuits in the cell as well as the magnetic field distribution with cracks.

### Flawless cells

**Figure 3:**
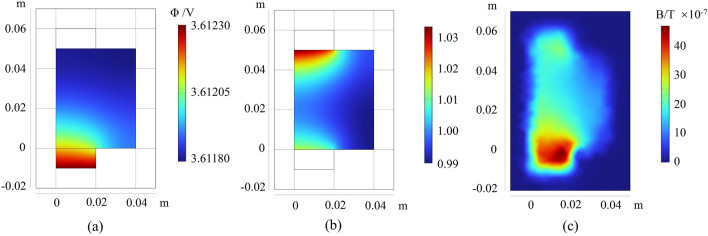
(**a**) Voltage distribution in the positive current collector of a flawless battery during the charging state. (**b**) Relative current density distribution in the separator of a flawless battery during the charging state. (**c**) Magnetic field distribution diagram of a fault-free battery during the charging state.

First, we conducted calculations for the potential distribution, current density distribution, and surrounding magnetic field of a healthy battery. Figure 3 illustrates the potential, relative current density, and magnetic field distribution during the charging process under 4 V 1C condition. As in Fig. 3a, a non-uniform voltage distribution within the current collector is clearly observed. The voltage near the ground terminal is lower than the average voltage, while the voltage near the input ground terminal is higher than the average voltage. Consequently, the non-uniform voltage distribution results in a non-uniform distribution of current during the charging process.

The simulated results of current density distribution of the flawless cell at the middle of the separator at the beginning of the 1C charge are illustrated in Fig. 3b. In the design of lithium-ion battery pouch cell, all current exits the cell on the cell “tabs”, resulting in higher current density near the positive and negative electrode tabs. As the charging process progresses, the current density in the central portion of the cell increases. The non-uniformity in current density distribution leads to an uneven distribution of the magnetic field within the cell.

As in Fig. 3c, for the charging condition of 4 V 1C, the magnetic field component images on the upper air domain surface are presented at the beginning of cell charging. According to the Biot-Savart law, the distribution of the magnetic field depends on the distribution of the current. As can be seen that due to the instability of the current, the cell will generate a corresponding magnetic field in the surrounding space. Since the current flows from the positive collector, the current density is higher at the collector, and accordingly, the magnetic field is higher here. From the simulation results, it is evident that during normal battery operation, the magnetic field generated by the current on the designated air domain surface ranges approximately from 1 to 4 μT, with a maximum reaching 4.6 μT.

### Internal short circuit

Battery thermal runaway refers to the phenomenon of rapid overheating within a battery characterized by an exothermic chain reaction occurring internally, leading to a drastic change in the rate of temperature rise^34–36^. When a battery experiences a short circuit due to external impact or internal faults, the Ohmic heating generated by the short-circuit current leads to overheating of the battery, resulting in thermal runaway^37,38^. Internal short circuit refers to the phenomenon where the separator within a battery is damaged or compromised, resulting in a direct connection between the positive and negative electrodes of the battery. The primary causes of internal short circuit faults are threefold: electrical abuse, mechanical abuse, and thermal abuse^39^. Mechanical misuse and thermal misuse can be mitigated through the implementation of standardized battery production and usage practices, whereas electrical misuse cannot be entirely avoided through these means. Electrical misuse is primarily induced by short circuits, overcharging, or over-discharging. In the case of a short circuit, the discharge current of the battery is substantial, generating a significant amount of polarization heat and Ohmic heat, resulting in the melting of the separator and the establishment of an internal short circuit by connecting the positive and negative electrodes. Overcharging and over-discharging, on the other hand, lead to the deposition of lithium and copper on the negative electrode^40,41^. The accumulation of lithium and copper forms metal dendrites, which, with continued reaction, may penetrate the separator, causing an internal short circuit within the battery.

**Figure 4:**
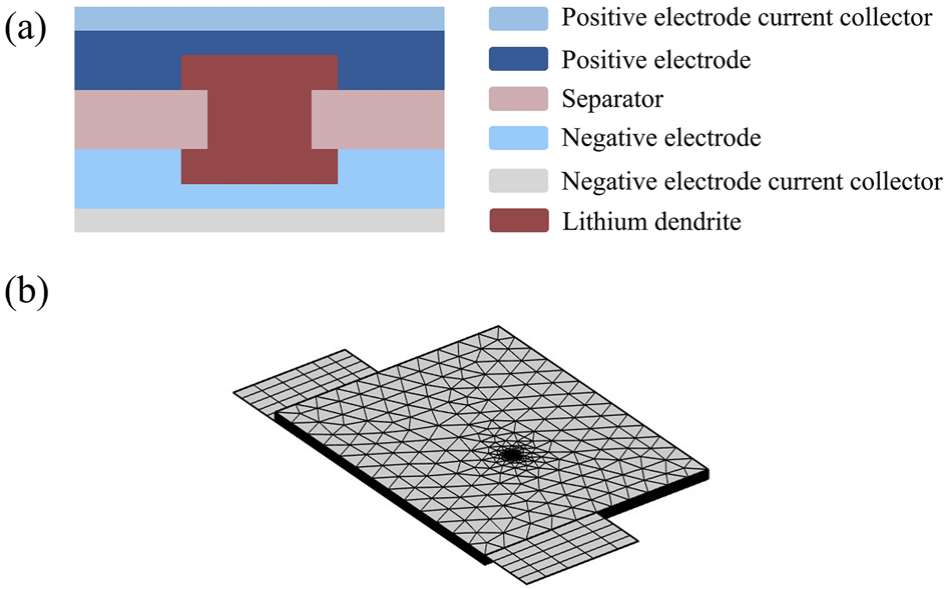
(**a**) Schematic diagram of the internal short circuit of the LIB caused by the penetration of lithium dendrites through the separator. (**b**) The grid division diagram when internal short circuit is caused by dendrites.

Based on the lithium-ion cell model established in the preceding section, this section introduces a short circuit point in the physical model and constructs a coupled multiphysics model electrochemistry-magnetic field-thermal coupling. The designated short circuit point is located at x = 0.02 m, y = 0.02 m, with a dendrite radius set at 50 μm. As shown in Fig. 4a, when an internal short circuit occurs in the battery, the positive and negative electrode materials are connected through the dendrite. It is assumed that the shape of the lithium dendrite is a cylinder with the same height as the separator in this work. At the same time, electrochemically inactive regions are set in both the positive and negative electrode domains to enhance the convergence of the model. The schematic of the mesh partition for the model is presented in Fig. 4b. Due to the significant temperature and magnetic field gradient changes in the short circuit region, the mesh is refined in the corresponding areas.

**Figure 5:**
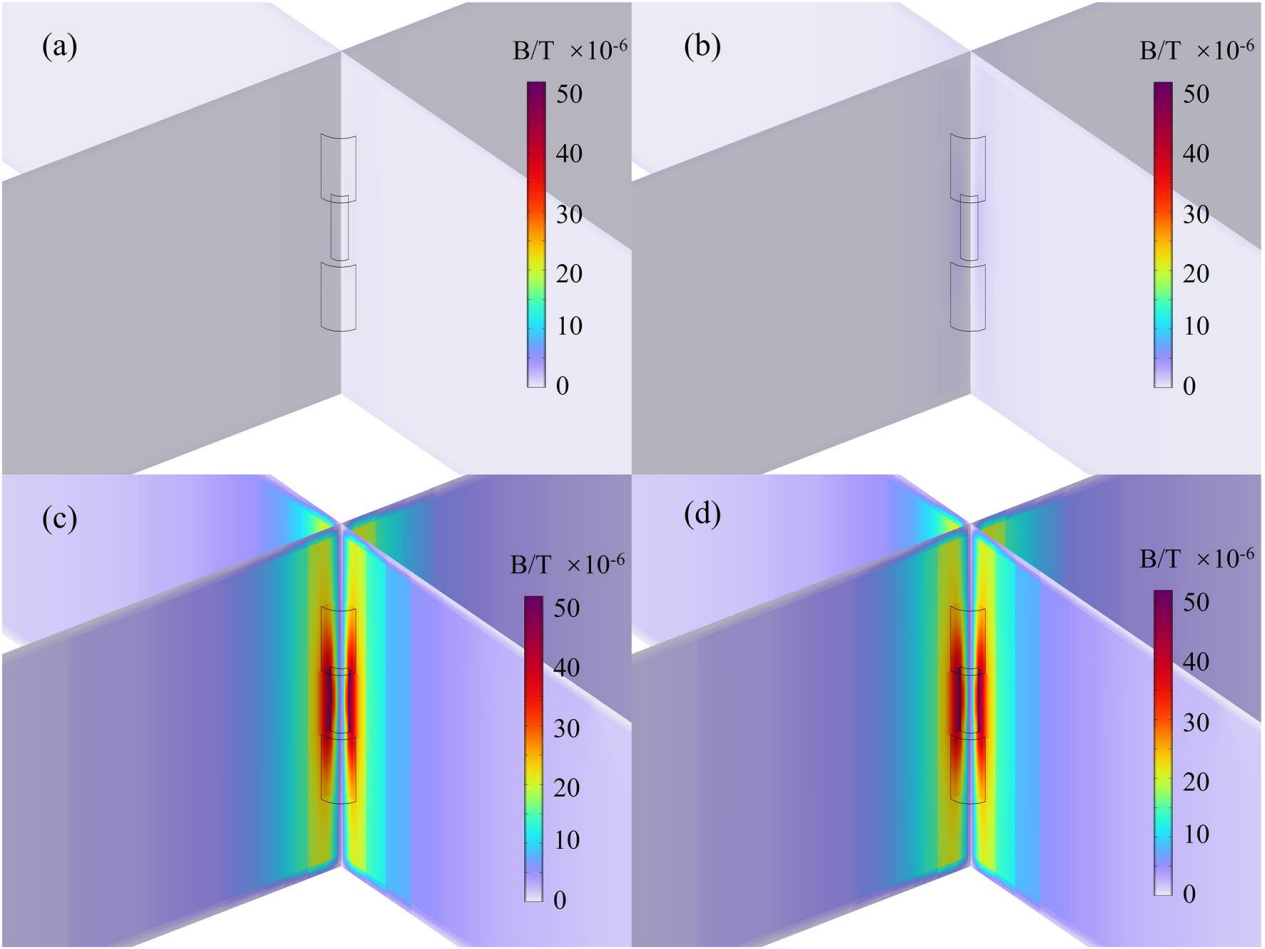
The simulated distribution of regional magnetic field near lithium dendrites at different time (50 μm): (**a**) 0 s; (**b**) 0.001 s; (**c**) 0.01 s; (**d**) 0.1 s.

Figure 5 presents simulation results of the magnetic field distribution around the short-circuit region in a battery with 50 μm dendrites at 0 s, 0.001 s, 0.01 s, and 0.1 s following the occurrence of an internal short circuit within the battery’s 5-layer cells. Given that the dendrite radius is significantly smaller than the geometric dimensions of the battery, the magnetic field distribution in the dendrite region is magnified for ease of observation. From the figures, it is evident that in the vicinity of the lithium dendrite, there is a pronounced increase in the magnetic field. Simultaneously, the highest magnetic field is observed at the dendrite position, and the magnetic field variation is confined to a small space near the dendrite. In a normally operating battery, internal current flows from the negative electrode to the positive electrode. As the lithium-ion battery undergoes charging and discharging cycles during the electrochemical reactions within the liquid electrolyte, excess lithium ions combine with electrons transported from the negative electrode when the embedded lithium content in the graphite exceeds its capacity. Due to factors such as uneven current density and lithium-ion distribution, lithium ions unevenly deposit on the surface of the negative electrode, forming lithium dendrites. Once the lithium dendrite grows to a certain extent, it can penetrate the separator, causing an internal short circuit in the battery^42,43^. At the onset of internal short circuit, the current flows along the lithium dendrite from the positive electrode to the negative electrode. The lithium dendrite acts as a conductor, resulting in a significant abnormal increase in short-circuit current. Correspondingly, the magnetic field at the short-circuit location also exhibits an abnormal increase.

**Figure 6:**
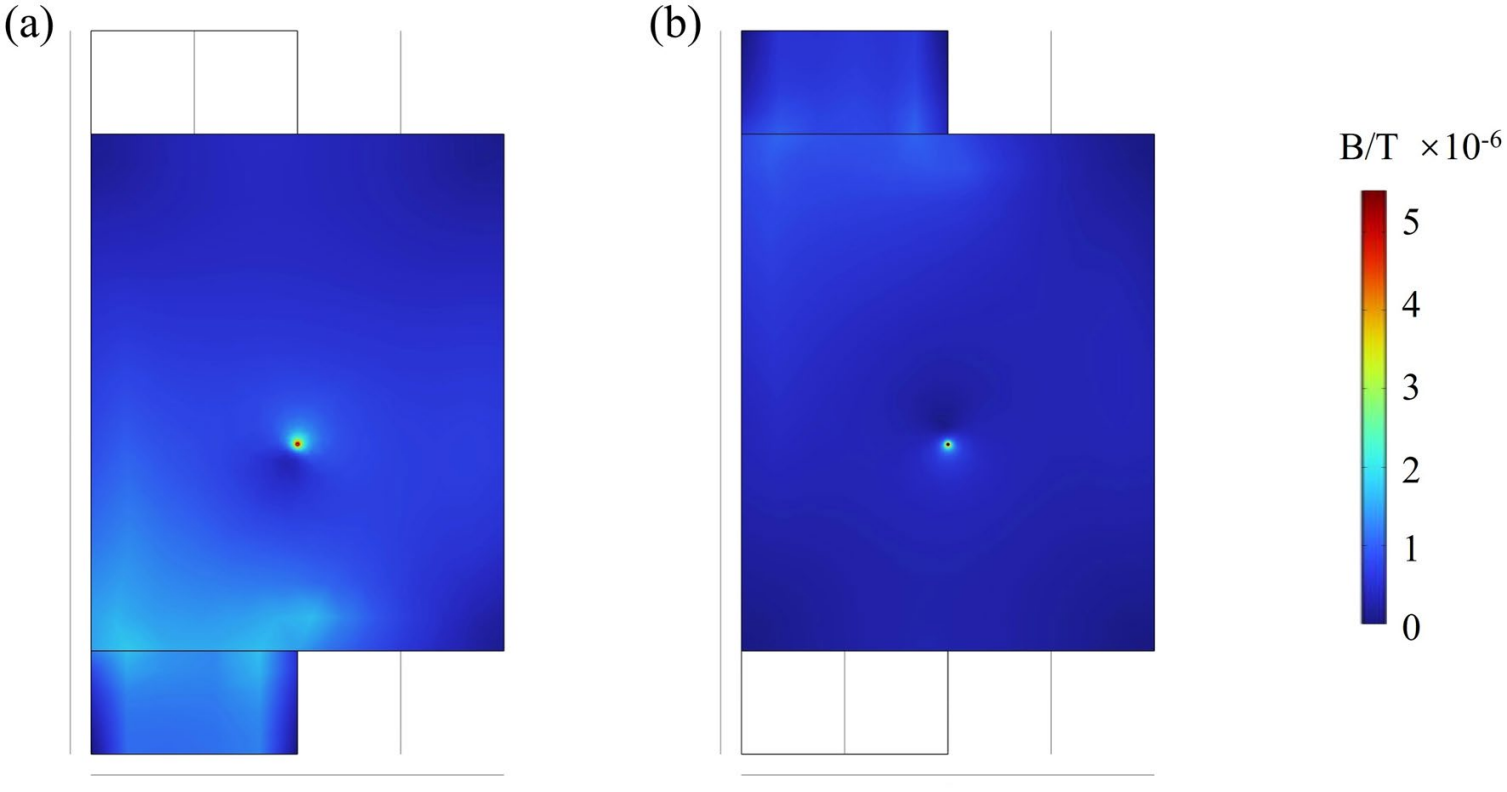
The magnetic field distribution on the surface of the battery at 0.01 s following the short circuit: (**a**) positive electrodes; (**b**) negative electrodes.

The magnetic field distribution images on the surfaces of the positive and negative electrodes are illustrated in Fig. 6. At 0.01 s after the short circuit, a distinct phenomenon of increased magnetic field is clearly observable on the battery surface, particularly at x = 0.02 m, y = 0.02 m, where the anomalous magnetic field value is approximately 5 μT larger than in other regions. In the operational lifespan of commercial batteries, internal short circuits are not directly observable from the outside. However, this phenomenon allows us to employ magnetic flux sensors and other magnetic measurement devices for battery diagnostics. By detecting the abnormal increase in the magnetic field, the number of fault points can be determined, and the fault locations can be identified to ensure the safe use of the battery.

**Figure 7:**
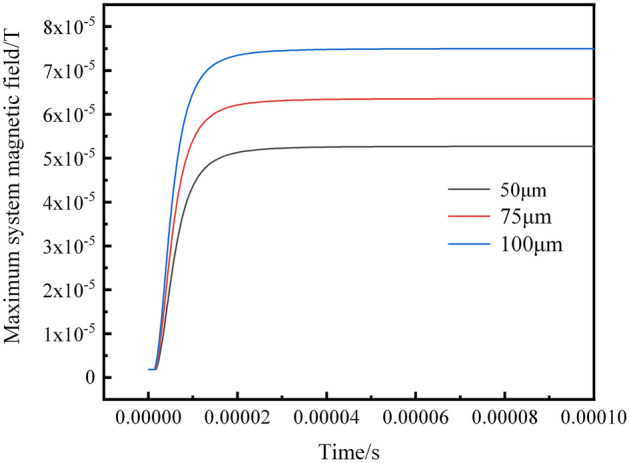
The maximum magnetic field of the simulated system for different dendrite radius.

Figure 7 shows the results of the maximum magnetic field values in the system after the occurrence of internal short circuits induced by lithium dendrites with different radius. The dendrites can be considered as cylindrical lithium metal conductors with constant material properties and height. The resistance of the dendrites decreases as the base area increases, leading to an increase in short-circuit current with the growing radius of the lithium dendrites. According to the Biot-Savart law, as the current in the short-circuit region increases, the corresponding magnetic field values also increase. When the dendrite radius is 50 μm, the maximum magnetic field value in the system is approximately 55 μT. When the dendrite radius increases to 100 μm, the maximum magnetic field value in the system reaches about 75 μT. Therefore, by detecting the magnetic field values of the battery, we can not only determine the position of the dendrites but also estimate the size of the dendrite radius. It is worth noting that, due to the magnitude of the maximum abnormal magnetic field values being comparable to the Earth’s magnetic field (approximately 50–60 μT), under the measurement conditions where the Earth's magnetic field serves as the background field, a clear distribution of abnormal magnetic fields can also be detected.

**Figure 8:**
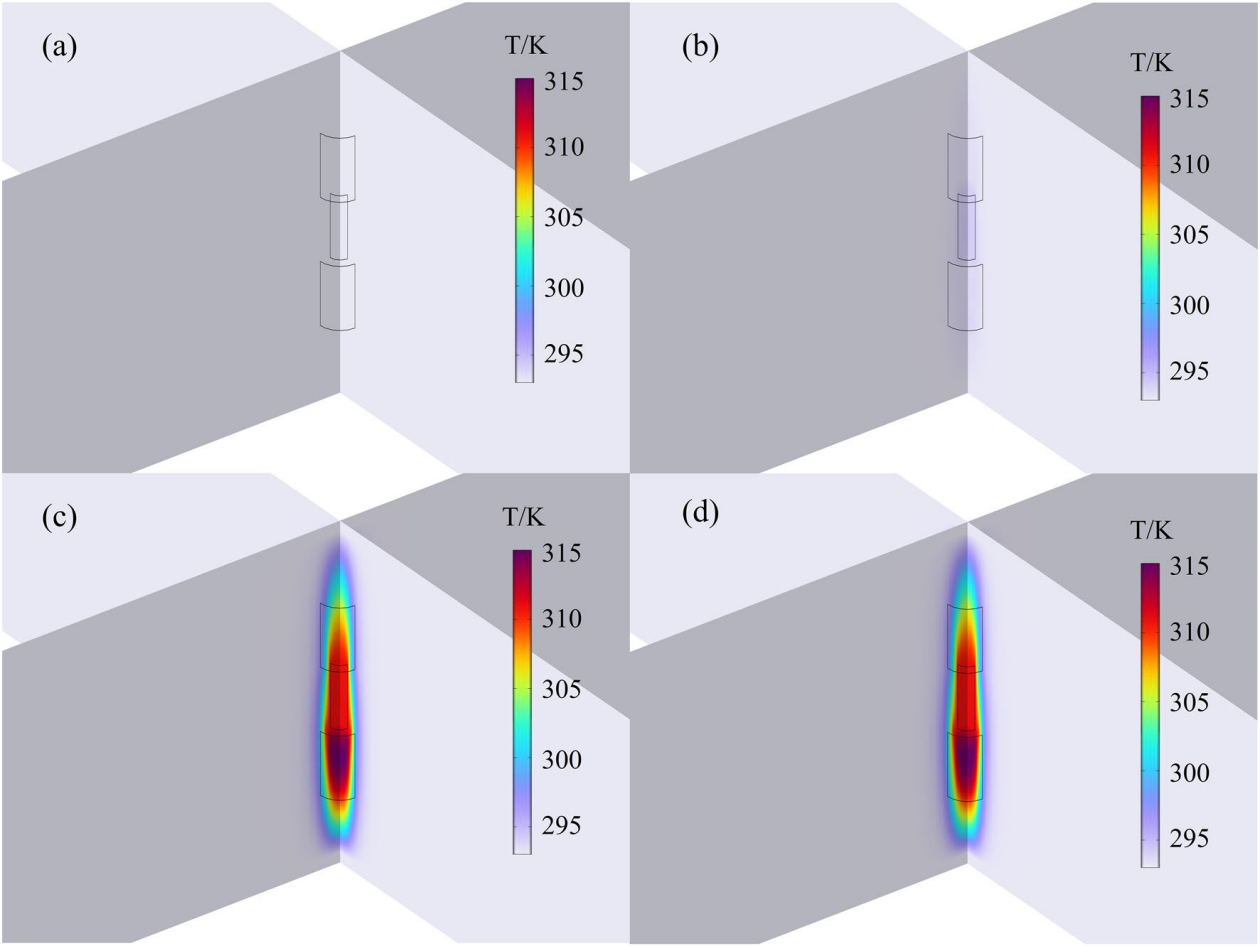
The simulated distribution of regional temperature near lithium dendrites at different time (50 μm): (**a**) 0 s; (**b**) 0.001 s; (**c**) 0.01 s; (**d**) 0.1 s.

The temperature elevation in the battery structure is directly correlated with the internal flow of current. Therefore, the lithium dendrites exhibit an initial temperature increase, and the surrounding structure gradually experiences elevated temperatures with the lithium dendrite at the center. The primary sources of heat generation during the internal short circuit process include reversible entropy heat and irreversible polarization heat from electrochemical reactions occurring on the positive and negative electrodes, Joule heat generated by lithium ions embedding and stripping within the positive and negative electrodes, and Joule heat generated when electrons pass through the positive and negative current collectors and the lithium dendrite^44,45^. The result of temperature distribution in the 5-layer cells near the short-circuit region is illustrated in Fig. 8. At 0 s, the initial temperature of the lithium dendrite is the same as the ambient temperature, which is 293.15 K. By 0.001 s, the highest temperature near the lithium dendrite rises to approximately 295 K. At 0.01 s, the maximum temperature in the short-circuit point area reaches around 315 K. Following a short circuit fault in the battery, the maximum temperature is achieved within 0.1 s, reaching approximately 315 K. Additionally, it can be observed that the temperature rise area of the negative electrode material is significantly larger than the corresponding area on the positive electrode material. This is attributed to the lower specific heat capacity of the negative electrode. Correspondingly, within the time frame of 0.1 s, the system’s highest temperature occurs in the region near the negative electrode surface adjacent to the lithium dendrite.

During an internal short circuit of a battery the two electrode materials are internally interconnected electronically, leading to a localized increase in the current density. This results in excessively high local temperatures and the consequential damage to the battery. In severe cases, it may even lead to fire incidents. The abnormal distribution of current can cause an anomalous distribution of the magnetic field, making fault diagnosis of internal short circuits feasible through magnetic field detection methods. Another commonly used approach by scientists in the study of internal short circuit faults is the temperature detection method. However, due to the relatively slow temperature response, measurements may introduce inaccuracies^13^.

**Figure 9:**
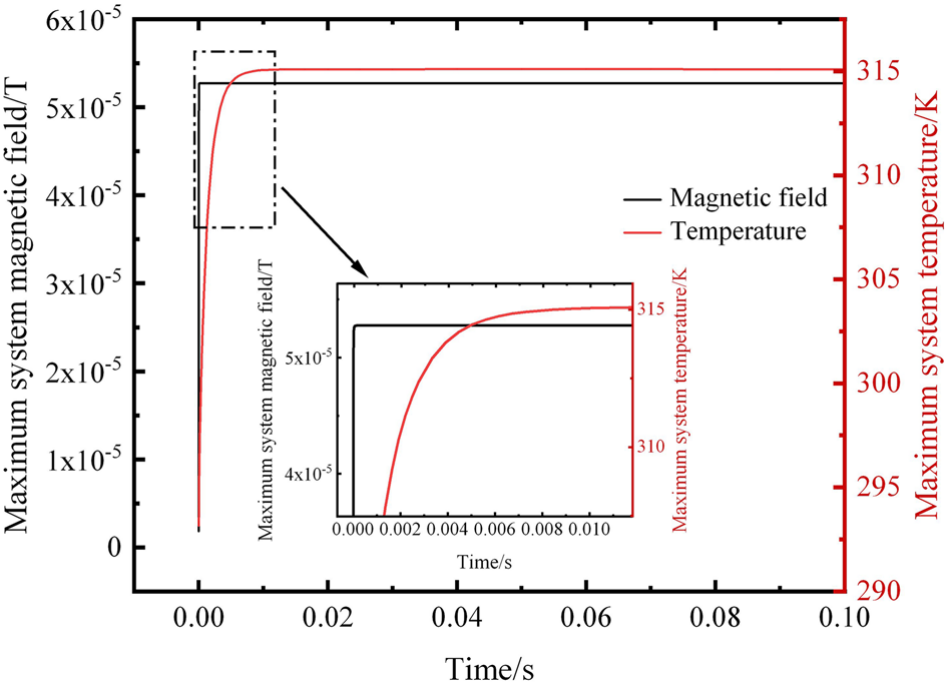
Variation of the maximum magnetic field and maximum temperature over time.

The variation over time in the maximum magnetic field values and maximum temperature values of the simulated system is shown in Fig. 9. The results evident that within an extremely short time after the occurrence of a short circuit fault, both the maximum magnetic field and temperature values in the system experience a rapid increase. The magnetic field response precedes the temperature response, with the maximum value of the system’s magnetic field reaching approximately 5.25 × 10^-5^ T in less than 0.001 s, after which the system's maximum magnetic field value changes tend to stabilize. In contrast, the system's maximum temperature value takes about 0.1 s to reach 315 K and then stabilizes. Therefore, under internal short circuit conditions, using magnetic field detection for state monitoring and health assessment of the battery is a superior method.

### Cracks (or cuts, scratches)

In the manufacturing process of batteries, potential issues such as cracks may arise from improper welding or instances of compression and impact during usage. After sealing or formation, it is difficult to obtain spatial resolved data of the battery from the outside. Utilizing magnetic field distribution maps with cracks allows for an intuitive assessment of the crack's condition, facilitating fault detection and precise crack localization.

**Figure 10:**
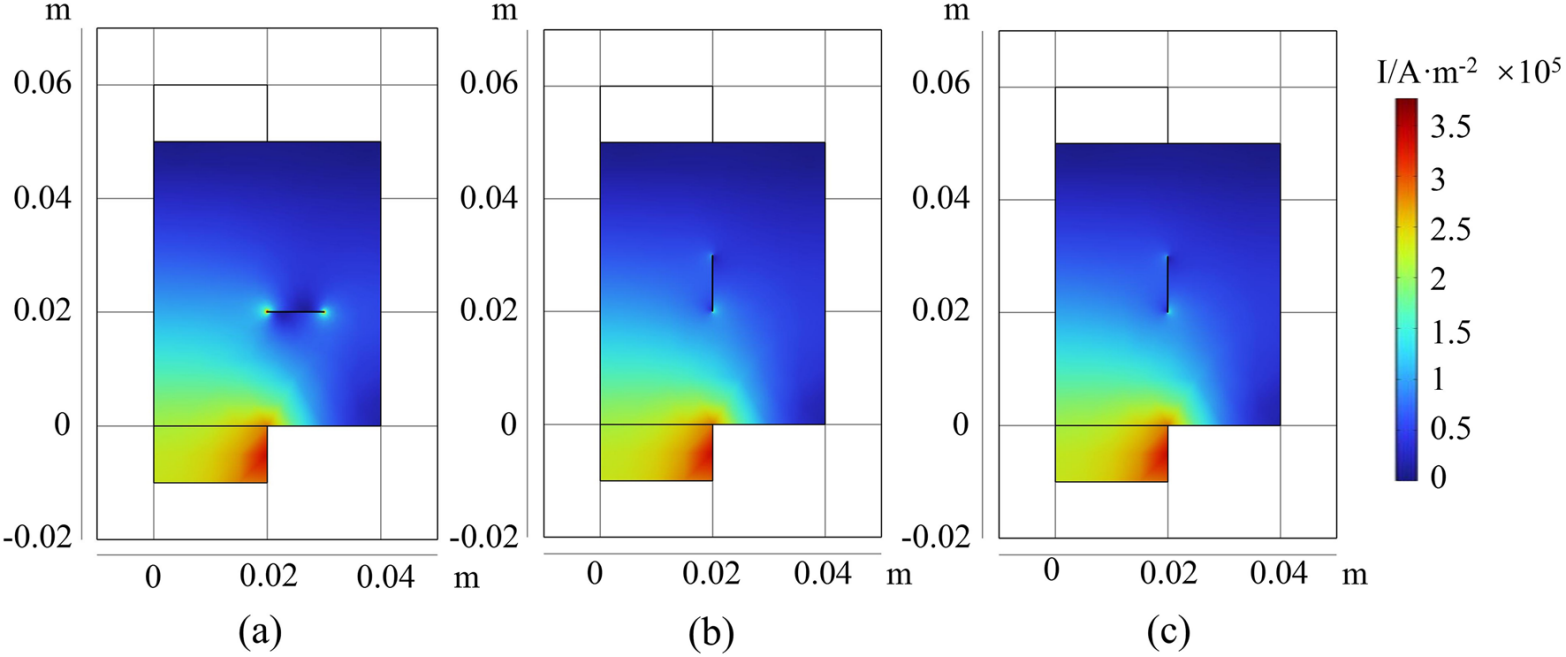
The current distribution of the battery when there are cracks in different directions: (**a**) along the x-axis; (**b**) along the y-axis; (**c**) At a 45° angle to the x-axis.

Figure 10 shows cracks in different orientations in the lithium-ion battery. The first scenario involves a crack parallel to the X-axis located at x = 2.5 cm, y = 5 cm, with a depth of 65 μm. As evident from the current density distribution diagram, the presence of a surface current from negative to positive in the xy plane causes the current to "circumvent" the crack in the battery, resulting in higher current density at both ends of the crack.

**Figure 11:**
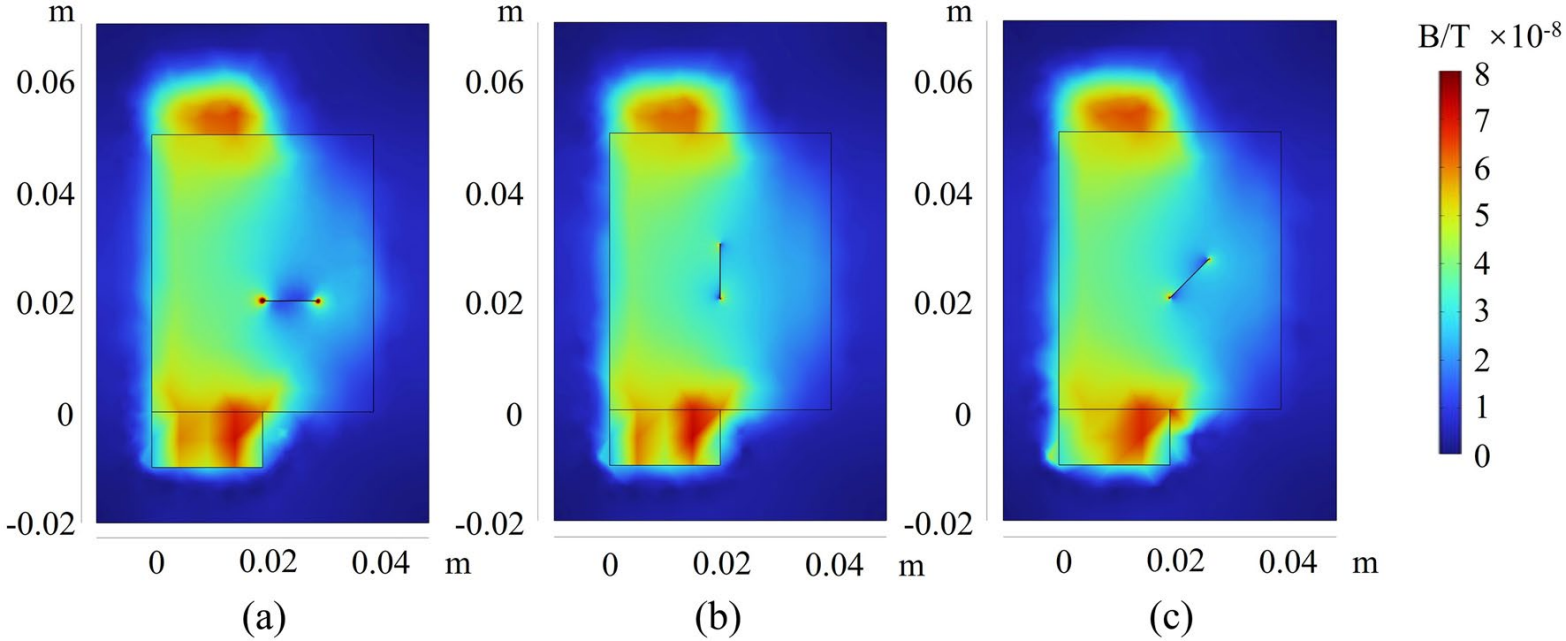
Magnetic field distribution of the battery when there are cracks in different directions: (**a**) along the x-axis; (**b**) along the y-axis; (**c**) At a 45° angle to the x-axis.

For encapsulated batteries, obtaining current density distribution images from the exterior is challenging; however, magnetic field information can be easily detected through magnetic sensors. Regarding the magnetic field distribution of the battery, results similar to current distribution can be observed. As shown in Fig. 11, the magnetic field images around a battery with a crack are displayed. Apart from significantly elevated magnetic field values at the tabs, there are also abnormally high magnetic fields at both ends of the crack. Additionally, there are conspicuous gaps in the magnetic field image at the crack’s site. Through the magnetic field distribution images, the locations and quantities of cracks in the battery can be easily detected. For the charging condition of 1C, the abnormally high magnetic field values are approximately 8 × 10^-8^ T. From Fig. 11a, it can be noted that the abnormal magnetic field values at the two ends of the crack are different. The abnormal magnetic field values are stronger at x = 0.02 m than at x = 0.03 m. This is because x = 0.02 m is closer to the tabs of the battery, resulting in a relatively larger current flowing through this region. In addition, in Fig. 11a, the current and magnetic field at both ends of the crack are greater than in Fig. 11b and c. This is because the direction of the crack in Fig. 11a (in the x-direction) is perpendicular to the flow direction of the current in the current collector (in the y-direction). Therefore, the current needs to “circumvent” the incision at both ends, leading to two positions with abnormally increased magnetic fields.

## Experimental verification

In practical applications, the nominal voltage and rated capacity of individual batteries are often insufficient to meet the demands of high-power usage. Thus, single batteries are commonly combined to form battery packs. During the configuration process of these packs, a prevalent approach involves initially parallel connecting individual batteries to create smaller battery pack units. Subsequently, these smaller units are arranged in series to construct larger battery packs. This hierarchical configuration method is designed to enhance the overall stability of the battery pack during operation.

Parallel operation effectively equalizes voltage and current discrepancies among individual batteries, thereby enhancing the performance consistency of the battery pack. Meanwhile, series operation facilitates voltage superposition to cater to scenarios requiring higher energy demands. Consequently, this parallel-then-series configuration method holds significant application value in battery system design.

However, over time and with repeated cycles, the performance consistency of batteries within the pack tends to deteriorate. Research indicates that variations in capacity or internal resistance directly contribute to uneven current distribution in parallel battery packs, accelerating battery aging and diminishing overall pack performance. Moreover, low-capacity batteries may induce abnormally high currents, generating excessive heat and posing potential safety hazards^46^.

The power banks represent a ubiquitous application of battery packs in daily life. Typically, these banks comprise single batteries arranged in series, parallel, or series–parallel combinations. A dedicated circuit board governs and manages the charging and discharging processes of the batteries, ensuring each operates within a safe range. In commercial power banks, the closure of the entire battery pack due to a single battery malfunction often impedes prompt troubleshooting of the faulty unit.

Based on the background, to further validate the efficacy of the model developed in this study for practical applications, this section will conduct detailed measurement procedures on an 18650-battery pack. However, acquiring faulty batteries for experi-mentation poses significant challenges. Manual disassembly or fault analysis, such as through nail penetration test, not only demands professional instrumentation and equipment but also entails inherent safety risks. Particularly with commercial power banks, unauthorized dismantling without proper equipment greatly heightens the risk of severe safety incidents, including fire and explosions.

Considering these factors, the experimental design of this section adopts an innovative approach: within a four-cell compartment, batteries will be selectively removed from different locations to simulate single-cell failures within the battery pack. Furthermore, when the battery has a fault such as short circuit or particle rupture, it will produce a weak magnetic field change. This change, while not easy to detect, can be detected with sensitive magnetic field sensors. For example, fluxgate can detect magnetic field changes of ~ nT magnitude^47^; and the SERF atomic magnetometer can detect magnetic field changes of the order of 10fT^48^. This method allows us to effectively replicate the fault conditions of the battery pack in real-world usage scenarios while prioritizing safety. Subsequently, precise measurements will be conducted on both the intact battery pack and the simulated fault state to comprehensively assess the model’s performance in practical settings.

### Experimental setup

When the battery is in operation, its magnetic field undergoes fluctuations during the charging and discharging processes. A magnetic sensor is employed to capture the magnetic field variations induced by the battery. This section utilizes scanning techniques to gather magnetic field data. Specifically, a fluxgate probe (with a measuring range of ± 70 μT and frequency domain noise less than 5pT/Hz^1/2^@1 Hz) is mounted onto a linear module sliding table, which moves around the battery during its operation to capture the magnetic field distribution within a defined plane.

For data collection, the fluxgate is fixed onto the holder, and the linear translation stage is connected to the bracket, enabling movement and scanning. The spatial position of the probe can be adjusted and calibrated manually. The stepper motor and controller integrated with the linear module enable precise control over its motion, facilitating the fluxgate’s scanning measurements.

**Figure 12:**
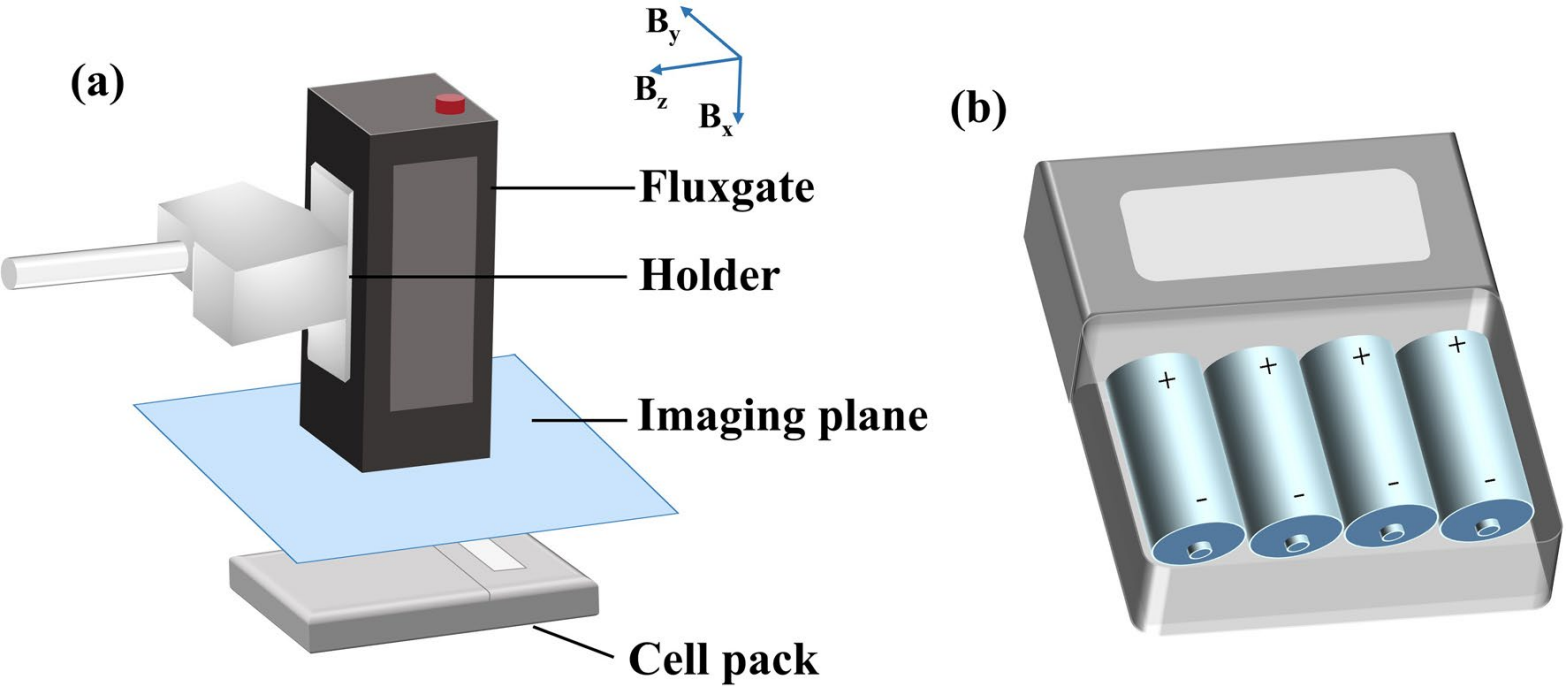
Experimental setup: (**a**) Arrangement of measurement setup. (**b**) Diagram of the power bank used in the experiment.

The reference coordinate system utilized during measurement is defined by the three-axis measurement coordinate system of the fluxgate, as illustrated in Fig. 12. (a). The blue arrow denotes the three-axis component measured by the fluxgate. When assessing the magnetic field distribution surrounding the battery, the y–z plane is designated as the scanning plane, and the fluxgate gathers magnetic field data based on its preset parameters. The scanning step length is 12 mm, with a stabilization time of 3 s, and each data point's collection duration is set to 3 s. Following the stabilization period, the software of the three-axis fluxgate samples and stores the magnetic field data. The magnetic field value of each point is determined by averaging the data collected within 3 s. This process is iterated across the designated scanning plane, with the resultant magnetic field values correlated with their corresponding coordinates to generate a magnetic field distribution map within the scanning plane.

The diagram illustrating the power bank utilized in the experiment is depicted in Fig. 12b. It can be placed inside 1–4 batteries, and the connection between the batteries is in parallel. The internal working battery of the battery box is a commercial 18650 battery with a rated capacity of 2.5 Ah. Throughout the experiment, the battery testing system (Neware, BTS-20V10A) was initially employed to regulate the charging and discharging states of the battery, followed by the utilization of the magnetic field acquisition module for scanning and data collection. During the measurement process, the distance maintained between the fluxgate probe and the surface of the power bank was 4 cm.

### Experimental results

**Figure 13:**
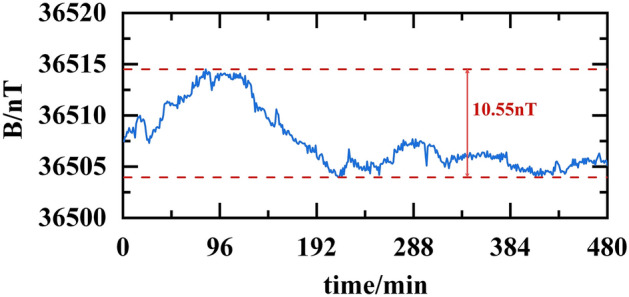
Fluctuations of the ambient magnetic field (continuous measurement for 8 h).

In lengthy and high-precision magnetic field measurement experiments, the inherent fluctuations of the Earth's magnetic field and the electromagnetic radiation emitted by laboratory equipment pose significant challenges to data accuracy. To ascertain the authenticity and precision of experimental data and assess the impact of background magnetic fields on results, we initiated an investigation. Initially, the fluxgate sensor was positioned within the laboratory’s geomagnetic environment and subjected to stable measurements over an uninterrupted period of 8 h, meticulously documenting the outcomes. As illustrated in Fig. 13, our findings revealed that while the background magnetic field exhibited fluctuations of approximately 10.55nT throughout the measurement period, these fluctuations were substantially smaller in magnitude compared to the targeted magnetic field range pertinent to the experiment. This revelation underscores our ability to accurately capture experimental phenomena amidst the intricate backdrop of the geomagnetic field, thus facilitating the derivation of scientifically reliable conclusions. Consequently, we posit that the influence of background magnetic fields on experimental outcomes is negligible, obviating the necessity for conducting experiments within a magnetic shield cylinder. This discovery not only streamlines experimental setups and procedures, enhancing efficiency, but also confirms the viability of utilizing magnetic detection methods to assess battery status within the geomagnetic field.

**Figure 14:**
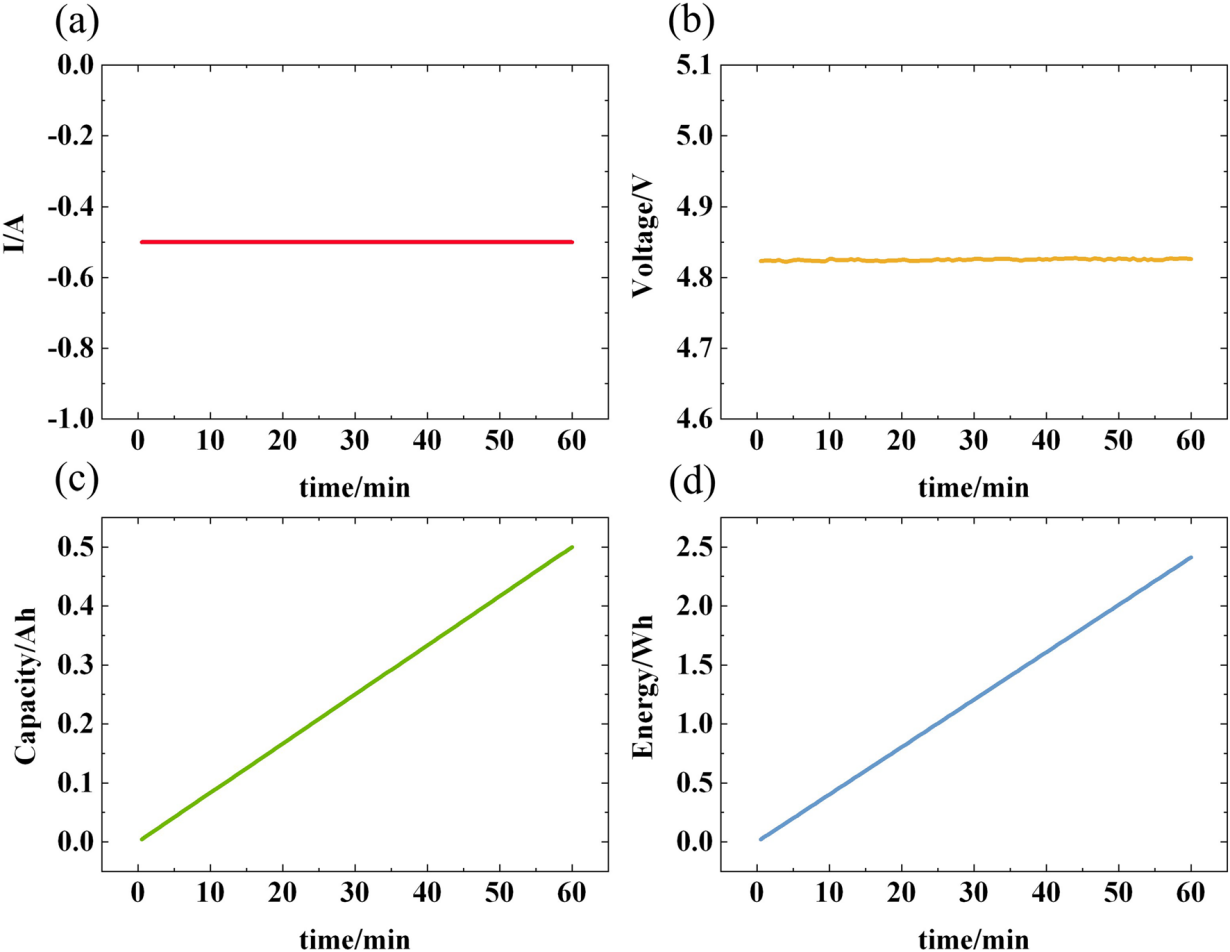
The results of battery tests: (**a**) The current; (**b**) The voltage; (**c**) The capacity; (**d**) The energy.

During the experiment, we employed the constant current discharge method to assess the battery pack, maintaining a consistent discharge current of 0.5A for a duration of 1 h. Figure 14 depicts the curves detailing the current, voltage, capacity, and energy of the tested battery pack over the course of charging and discharging. As illustrated, under the constant current discharge condition, the battery pack consistently maintains a current value of 0.5A, showcasing excellent current stability. Notably, the voltage of the battery pack remains nearly unchanged, hovering around 4.8 V at both the onset and conclusion of the discharge, indicating remarkable voltage stability throughout the discharge process. Given the parallel connection of batteries in this experiment, the output voltage remains steady even if individual batteries encounter issues. Furthermore, owing to the constant current discharge mode employed, the capacity and energy of the battery pack exhibit a linear correlation with time. This discovery furnishes a crucial experimental foundation for comprehending the performance dynamics of battery packs during constant current discharge, thereby offering valuable guidance for researchers seeking to delve deeper into battery performance optimization.

**Figure 15:**
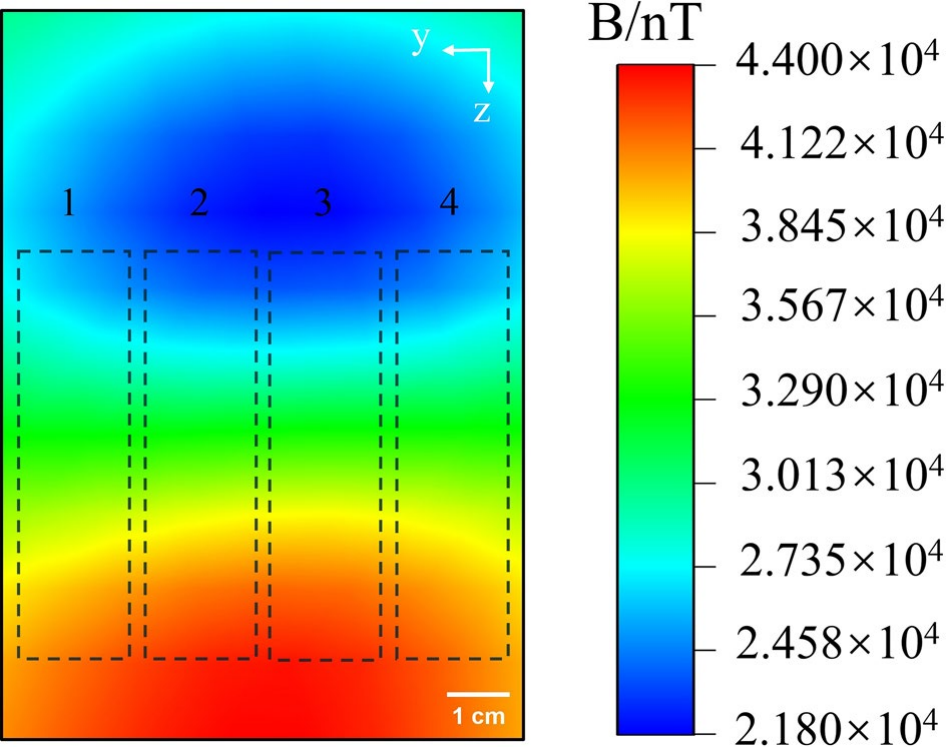
Magnetic field distribution when the healthy battery pack is discharged at 0.5A.

The magnetic field distribution image of the healthy battery pack during 0.5A discharge is depicted in Fig. 15, with each individual battery being marked with a corresponding number. Within the image, four black dotted boxes delineate the four battery slots within the box. Examination of the magnetic field distribution image reveals a degree of unevenness in the induced magnetic field distribution of the battery pack, attributable to the background field presence and the compounded effect of magnetic fields. Notably, as each individual battery undergoes discharge, current accumulation becomes apparent at positive and negative positions, leading to a discernible uneven distribution of magnetic fields in these areas. Additionally, the upper section of the image exhibits a more uniform magnetic field distribution compared to the lower portion. This disparity can be attributed to the upper part corresponding to the power bank’s monitor, where internal currents are smaller and more uniform, thus resulting in a more homogenous induced magnetic field distribution.

**Figure 16:**
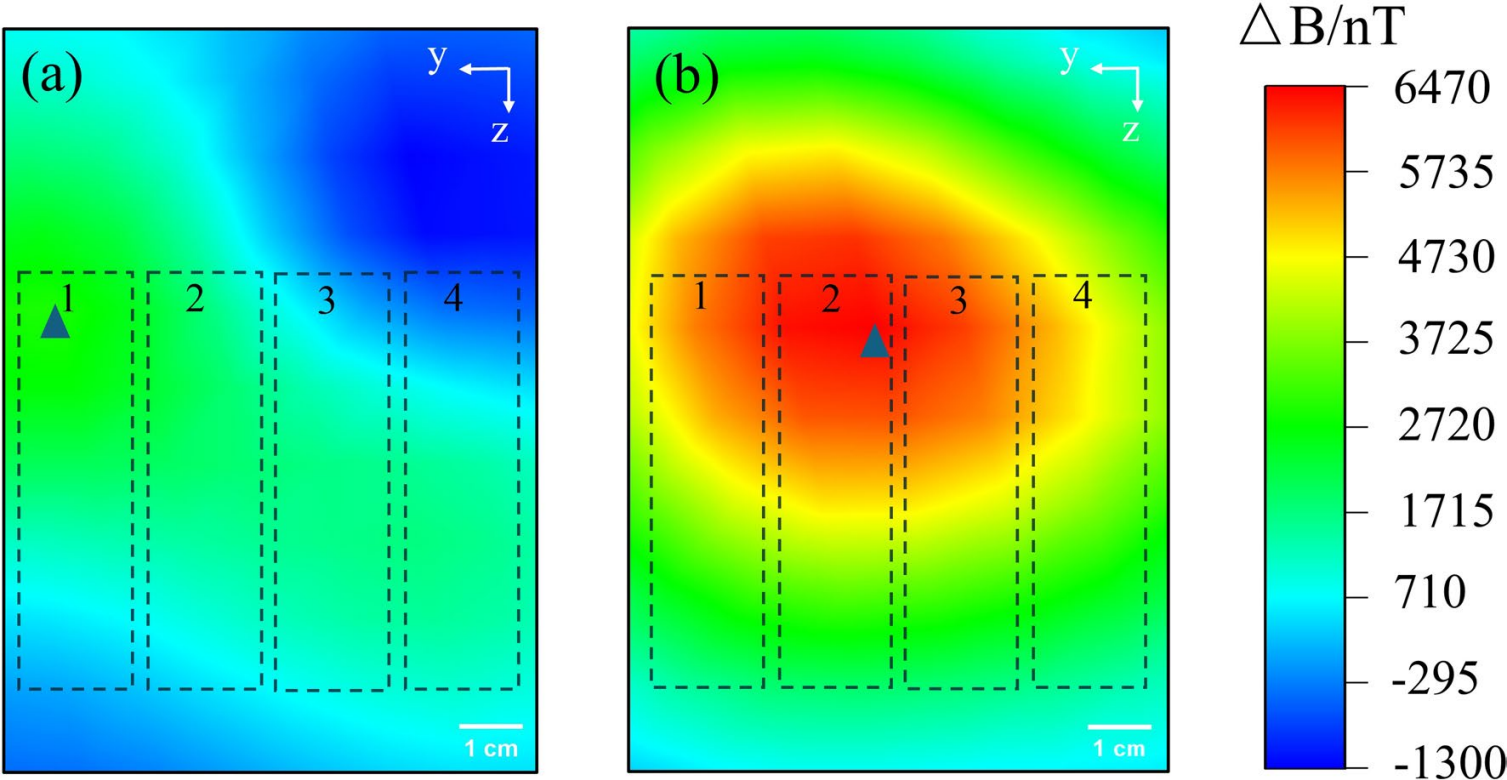
Magnetic field distribution when the battery pack is discharged at 0.5A: (**a**) The battery at position 1 fails. (**b**) The battery at position 2 fails.

The magnetic field of the power bank is too high, to the extent that it would cover the failed positions. To observe clearer experimental phenomena, we measured the magnetic field distribution, B2, of the faulty battery pack and compared it with the magnetic field value, B1, at the corresponding positions within a non-faulty battery pack to assess the magnetic anomaly ΔB. The resulting magnetic field distribution image is presented in Fig. 16. Experimental findings reveal that in the failure of the battery at position 1, a magnetic field anomaly ranging from approximately 2000–3000 nT manifests near the fault location, forming a circular magnetic field distribution image. The maximum magnetic anomaly value recorded is approximately 2934 nT, with its location corresponding to the number of the battery’s position, indicated by the blue triangle in Fig. 16a. Similarly, as depicted in Fig. 16b, when the battery at position 2 fails, a distribution pattern akin to that in Fig. 16a emerges near the fault location, with the magnetic field anomaly peaking at around 6735 nT.

In conclusion, the location of a failed battery within the power bank can be determined by identifying the location of the magnetic anomaly. This approach circumvents the need for monitoring battery pack capacity anomalies or reconstructing internal current images to pinpoint faults, thus enabling precise identification of failed battery locations.

Similarly, this method can also detect defective batteries when the internal current of the battery pack increases abnormally. If some batteries within the pack experience an internal short circuit leading to an abnormal rise in current, this deviation in current triggers an abnormal increase in the magnetic field surrounding the battery pack. Consequently, this method can detect the abnormal magnetic field resulting from the abnormal current and concurrently identify the faulty battery. The abnormal current caused by an internal short circuit often induces an abnormal rise in battery temperature. When the temperature of a lithium-ion battery surpasses 90 °C, the interface film of the solid electrolyte begins to decompose, potentially triggering a chain reaction leading to thermal runaway^49^. Hence, it is imperative to promptly identify and investigate battery faults. However, during normal battery operation, temperature also rises, which may impede the detection of abnormal temperature, resulting in a delay in identifying anomalies. As outlined in Sect. 3.4.1, the magnetic field measurement method exhibits a faster response time compared to temperature measurement, enabling more timely detection and localization of battery pack faults.

## Conclusion

This paper establishes a coupled 3D multiphysics model for the lithium-ion battery pouch cell by integrating electrochemical, magnetic field, and thermal models. Numerical simulations are conducted to investigate the distribution of physical fields surrounding the cell. Based on this model, the paper calculates and analyzes the physical field distribution under conditions of internal short circuits and the presence of cracks. The results indicate that under internal short circuit conditions, there is a significant abnormal increase in the magnetic field near the short circuit point, with the magnetic field anomaly on the battery surface approximately 5 μT higher than in other regions. The primary sources of heat generation in the battery, stemming from lithium dendrites, are the positive and negative electrodes. The larger the radius of the lithium dendrites, the greater the maximum magnetic field value in the system. In the case of a cell’s crack, there is a noticeable abnormal increase in the magnetic field at both ends of the crack. Whether in the scenario of internal short circuits or the presence of the crack, the number and location of faults can be determined by the abnormal increase in the magnetic field. The key advantage of this method is the non-destructive detection of the battery's health state, avoiding material deformation caused by disassembly and similar methods. Furthermore, since the temperature response is delayed compared to the magnetic field, utilizing magnetic field detection allows for early identification of short circuit issues, facilitating timely intervention to prevent accidents resulting from short circuits. Additionally, a magnetic field scanning method is proposed for model verification. The results demonstrate that the magnetic field image can accurately reflect the location of faulty battery cells and facilitate their selection and localization.

In future research, enhancing the accuracy of the model will be a primary challenge and focus. During battery faults, the abnormal magnetic field generated within the fault area exhibits large magnetic field gradients. To improve the model’s precision, further refinement of the grid is required to obtain more realistic computational results. Simultaneously, integrating this model with an aging model in future studies can facilitate the prediction of the battery’s lifespan. The large size of existing fluxgate sensors limits the measurement accuracy of small size batteries. In the future, high-resolution devices such as optical fiber magnetic field sensors can be introduced to obtain finer magnetic field distribution images, providing accurate references for battery production, detection and screening. In addition, future research can utilize small magnetic sensor arrays to achieve multi-point real-time measurements and accurately reconstruct magnetic field images. These research outcomes will contribute to the exploration of the mechanisms behind battery faults, providing a theoretical foundation for non-destructive battery testing. Additionally, they offer reference methods for the safety assessment and health evaluation of commercial batteries.

### Supplementary Information

Supplementary Figures.

## Data Availability

Data will be made available on request. If someone wants to request the data from this study, please contact baixy26@mail2.sysu.edu.cn by email.
