# Supplementary material for: Three-dimensional electrochemical-magnetic-thermal coupling model for lithium-ion batteries and its application in battery health monitoring and fault diagnosis
Source: Sci Rep. 2024 May 11;14:10802. doi: 10.1038/s41598-024-61526-0 (PMC11088670; doi:10.1038/s41598-024-61526-0)
Supplement: Supplementary file 1 — Supplementary Figures. [file 41598_2024_61526_MOESM1_ESM.pdf]

Supplementary file

A non-destructive detection method for lithium-ion cells based on precision magnetic field measurements

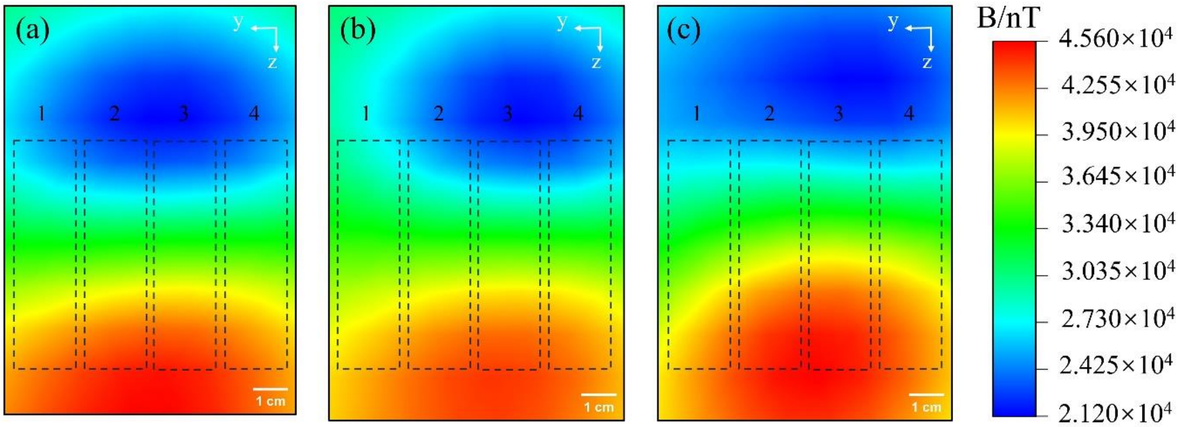

Supplementary figure 1. Magnetic field distribution when the healthy battery pack is discharged at 0.5A:

(a) No faults; (b) The battery at position 1 fails; (c) The battery at position 2 fails.

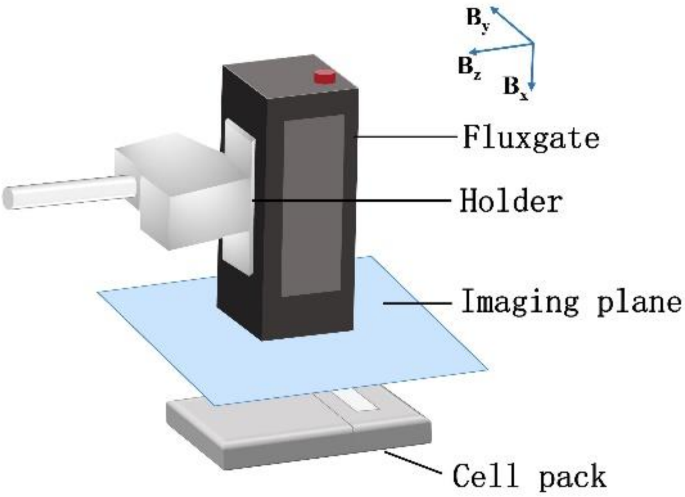

Supplementary figure 2. Arrangement of measurement setup.
